# Identification of Novel CSF-Derived miRNAs in Treated Paediatric Onset Spinal Muscular Atrophy: An Exploratory Study

**DOI:** 10.3390/pharmaceutics15010170

**Published:** 2023-01-03

**Authors:** Arlene M. D’Silva, Didu Kariyawasam, Pooja Venkat, Chelsea Mayoh, Michelle A. Farrar

**Affiliations:** 1 Department of Neurology, Sydney Children’s Hospital Network, Sydney, NSW 2031, Australia; 2 School of Clinical Medicine, Faculty of Medicine and Health, University of New South Wales, Sydney, NSW 2052, Australia; 3 Children’s Cancer Institute, Lowy Cancer Research Centre, UNSW Sydney, Sydney, NSW 2052, Australia

**Keywords:** spinal muscular atrophy, microRNA, nusinersen, pharmacodynamic, biomarker

## Abstract

The availability of disease modifying therapies for spinal muscular atrophy (SMA) have created an urgent need to identify clinically meaningful biomarkers that provide insight into disease progression and therapeutic response. microRNAs (miRNA) have been shown to be involved in the pathogenesis of SMA and have the potential to provide insight within the field of SMA. miRNA-sequencing was utilized to identify differential miRNA expression in the cerebrospinal fluid (CSF) in six children with SMA treated with nusinersen in this exploratory study. Fourteen differentially expressed miRNAs were significantly altered in CSF from baseline to follow-up during treatment with nusinersen. The greatest magnitude of change was noted in miR-7-5p, miR-15a-5p, miR-15b-3p/5p, miR-126-5p, miR-128-2-5p and miR-130a-3p which encompassed a spectrum of functions predominantly in neurogenesis, neuronal differentiation and growth. The dominant signaling pathways identified in this study were the mammalian target of rapamycin and the mitogen-activated protein kinase signaling pathways. This study identified multiple miRNAs that were involved in the complex interplay between neurodevelopment and neurodegeneration.

## 1. Introduction

Spinal muscular atrophy (SMA) is characterized by the degeneration of motor neurons in the anterior horn of the spinal cord and disruption of motor axon development in ventral roots due to the deficiency of the survival motor neuron protein (SMN) [1]. Homozygous deletions or mutations in the survival motor neuron 1 (*SMN1*) gene cause SMA [2]. The paralogous survival motor neuron 2 (*SMN2)* gene contains a single nucleotide change that produces alternative splicing with only low levels of functional SMN protein being formed [3]. *SMN2* can vary from 0 to 8 copies per genome and is the main modifier of SMA severity [4].

While SMN protein is ubiquitously expressed in the cytoplasm and nucleus of all cells [5], the selective vulnerability of motor neurons to cell death in SMA is not fully understood. SMN plays diverse roles in mediating a spectrum of molecular pathways, including modulation of DNA repair, RNA metabolism, cell signaling, autophagy and axon cytoskeleton structure. It is likely that SMN deficiency simultaneously disrupts a multitude of essential neuronal processes, contributing to disruption of motor neuron maturation, growth and survival in SMA [5]. In support of this concept, clinical and neurophysiologic studies demonstrate rapid motor decline during infancy, followed by slower progression among paediatric phenotypes. 

Therapeutic approaches for SMA focus on increasing the production of SMN protein. Nusinsersen (Spinraza^TM^) is an antisense oligonucleotide (ASO) that modifies the splicing of *SMN2* pre-mRNA to promote expression of functional SMN protein [6,7]. Intrathecal administration of nusinersen into the cerebrospinal fluid (CSF) facilitates its distribution to the central nervous system (CNS). The standardized administration regime involves four loading doses in the induction phase over two months followed by ongoing four monthly maintenance injections. Clinical neurophysiological studies have established the pharmacodynamic effects of nusinersen in children with SMA, with cessation of axonal loss, facilitation of peripheral motor axon development and increasing functional innervation of smaller motor units [8,9]. The molecular biomechanisms underpinning this therapeutic response are yet to be defined. 

In spinal motor neurons, microRNAs (miRNAs) are essential for the development, differentiation, acquisition and maintenance of axonal growth, cytoskeletal structure, synapse formation and overall activity [10]. miRNAs are a class of short single stranded non-coding RNAs that regulate the expression of more than one target gene simultaneously by pairing with their target mRNAs and contribute to the activity of interconnected signaling pathways. Although preclinical studies have shown dysregulation of miRNAs play a role in SMA pathogenesis, changes in expression with disease modifying therapy are essential to capture their potential as predictive, prognostic and pharmacodynamic biomarkers and have not yet been elucidated. miRNAs have the potential to answer outstanding clinical questions that are emerging within the field of SMA and facilitate a pathway driven integrated analysis of SMN interactions.

Increasingly, CSF diagnostics focusing predominantly on the proteome and metabolome are being developed to ascertain disease onset, progress and response to therapy in SMA [11]. However, these omic approaches have limitations in their use across the age, phenotypic and genotypic spectrum and may not be entirely disease specific. In contrast, the analysis of CSF miRNAs implicated in disease process and therapeutic intervention, may address prevalent knowledge gaps. Furthermore, to ascertain the effects of a CNS targeted therapy such as nusinersen, there is an urgent need to investigate miRNAs in the CSF. 

The primary aim of this exploratory study was to ascertain the feasibility of utilizing a low-input CSF transcriptomic profiling approach for the first time in childhood onset SMA. The secondary aim was to use an untargeted methodology and bioinformatic filtering approach to identify miRNAs modulated by nusinersen treatment, define their associated target genes and signaling pathways. 

## 2. Materials and Methods

### 2.1. Participants and Setting

This was a single-center prospective longitudinal study. This study included infants with SMA between December 2019 and January 2021 at the Sydney Children’s Hospital, Randwick, New South Wales for whom a CSF specimen was collected during the course of nusinersen treatment, administered as standard clinical care. The first specimen collected from the individual was deemed as baseline and compared to a follow-up sample, collected during the later course of nusinersen treatment. As this was a real-world study, there was variability in the timing and number of CSF collections between individuals. All children had genetically confirmed homozygous exon 7 *SMN1* deletions. We included children who were either diagnosed through newborn screening or after developing clinical signs and symptoms of SMA from birth to 1 year of age. Children who received sequential or combinatorial therapy with other disease modifying agents were excluded from this study. Parents of participants signed informed consents for the use of CSF and clinical data for research purposes. The study was approved by the Sydney Children’s Hospital Network Human Research Ethics Committee (HREC/18/SCHN/373). 

### 2.2. CSF Handling and RNA Isolation

**Figure 1:**
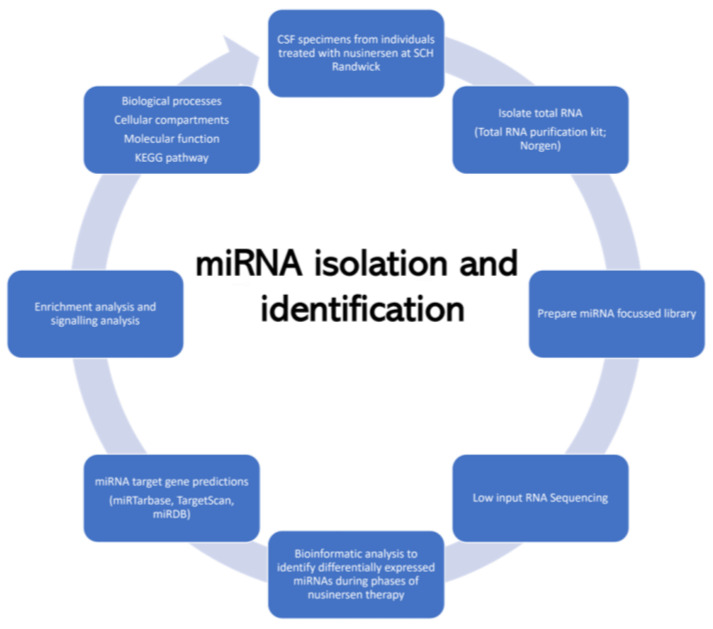
Flowchart of the experimental approach for identification of miRNA in cerebrospinal fluid of children affected with spinal muscular atrophy.

CSF samples were centrifuged at 500× *g* for 10 min at 4 °C and the supernatant were aliquoted to 1 mL Eppendorf tubes and stored at −80 °C till further analyzed (Figure 1). CSF samples containing visible blood cells after centrifuging were excluded. 

Total RNA purification kit from Norgen Biotek Corporation, Ontario, Canada (Product # 17200) was utilized to carry out RNA extraction. To prepare the cell lysate, 200 µL of CSF was transferred to an RNase-free microcentrifuge tube. Then, 600 µL of lysis solution containing was added to the CSF and vortexed for 10 s. Next, 800 μL of 96–100% ethanol was added to the lysate and was added to every 800 μL of the lysate and combined by vortexing for 10 s. To facilitate binding of the lysate to the column, the lysate with the ethanol was centrifuged for 1 min after which the flowthrough was discarded. The column was washed with 800 μL of 96–100% ethanol for a total of three times before the column was spun for two minutes to thoroughly dry the resin. RNA elution was carried out by adding 30–50 μL of elution solution to the column. The column was centrifuged for 2 min at 2000 revolutions per minute (rpm), followed by 1 min at 14,000 rpm. A repeat elution was carried out to increase the recovery of the RNA. The purified RNA sample was stored at −80 °C till further analysis. 

### 2.3. miRNA Expression Analysis

The quantity and quality of the isolated RNA were assessed by the Agilent 2100 Bioanalyzer™ (Agilent Technologies, Foster City, CA, USA) and by spectrophotometer using 260/280 nm and 260/230 nm ratios. CSF RNA concentrations and RNA integrity number were replicated to previously published literature, demonstrating the quality of the miRNA identified in our study. QIAseq miRNA library kit was used to prepare miRNA sequencing libraries (manufacturer: Qiagen). The QIAseq kit facilitates both enhanced differential expression analysis using integrated Unique Molecular Indices (UMIs) and novel discovery of miRNA from CSF. We analyzed miRNA expression from isolated RNA (range 9–12 ng/µL, 5 µL elute) by utilizing a low input RNA sequencing methodology (One NextSeq 1 × 75 bp High Output flowcell; to read miRNA plus the UMI). 

### 2.4. Bioinformatic Analysis and Integration of Transcriptomics with Genomics and Cellular Phenotypes

Processing of the raw fastq files and miRNA quantification was carried out using QIAGEN’s web-based analysis tool GeneGlobe (https://geneglobe.qiagen.com/us/analyze (accessed on 27 September 2021)). Raw data were aligned to the human reference genome (GRCh38) using GeneGlobe. miRNA UMI counts generated using the GeneGlobe tool were used to perform further downstream analysis. Differential expression analysis was performed comparing follow-up phase to the baseline phase using the R package edgeR [12,13,14]. Upper quantile and down sampling methods were used to normalize the miRNA expression data. By convention, a positive fold-change is indicative of the corresponding miRNA being up-regulated in the follow-up phase in comparison to baseline phase of therapy and a negative fold-change down-regulated. Differential miRNAs were identified if they were statistically significant with a *p*-value < 0.05 and a FC >= |2|. The significantly differential miRNAs underwent a systematic literature review and were further filtered for those miRNAs predominantly involved within motor neuron specific physiological and pathophysiological processes, as based on a comprehensive and systematic review of the literature. This filtering approach has been widely used in prior studies to focus transcriptomic profiling for miRNAs that are relevant to specific disease processes [15]. 

The target genes for differentially expressed miRNAs were predicted using mirRTarbase software and validated against the strength of the clinical and preclinical evidence for or against them (validation methods inherent to miRTarbase software) [16]. The top candidate target genes associated with strong evidence (based on reporter assay, Western blot or quantitative polymerase chain reaction methodology) linking the miRNA and its target gene were included in the subsequent analysis. Furthermore, the target genes for each differentially expressed miRNA were predicted using TargetScan [17,18] and miRDB [19] and only target genes supported by at least two independent tools were taken into consideration for further analysis. The suite of candidate target genes was then analyzed using the functional annotation clustering tool to identify their molecular, biological and cellular functions utilizing Gene Ontology (GO) and Kyoto Encyclopedia of Genes and Genomes (KEGG) pathways through the DAVID (Database for Annotation, Visualization and Integrated Discovery) gene annotation tool. We chose only pathways that had a false discovery rate (FDR) of <0.01. Significant functions identified at the GOTERM DIRECT level were analyzed further.

## 3. Results

### 3.1. Characteristics of the Study Cohort

This study included CSF samples from six infants with SMA, including a range of ages (neonatal period to late infancy at treatment initiation), disease duration, *SMN2* genotypes (2 and 3 copies) and disease stages (pre-symptomatic and symptomatic SMA) where symptomatic children were those who had signs and symptoms of SMA disease. Infants were followed for a median of 6 (range 1.6–11.7) months post dosing with nusinersen. All children attained motor function with nusinersen therapy (Table 1).

### 3.2. miRNA Levels Are Dynamically Modulated in SMA Patients Following Treatment with Nusinersen 

**Figure 2:**
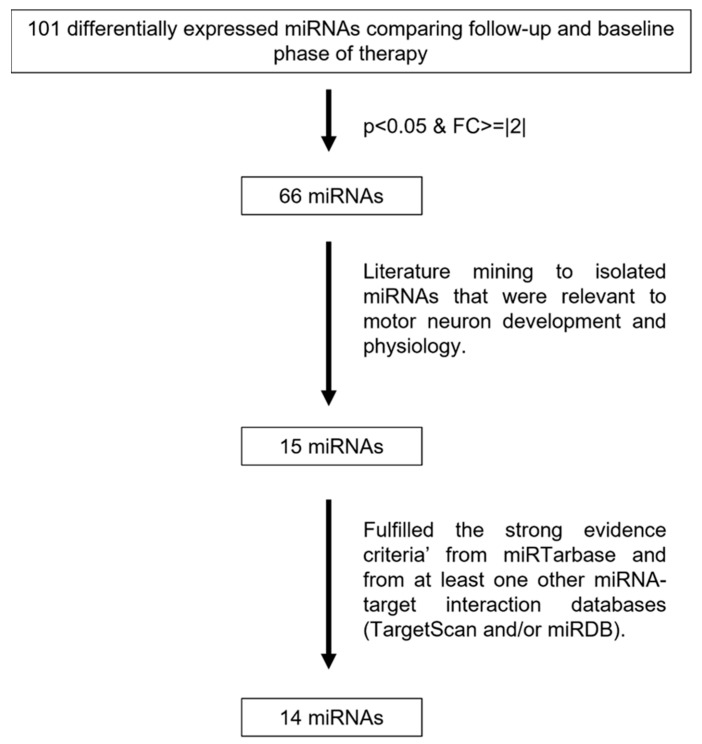
Flowchart depicting experimental approach for filtration of significant miRNAs in cerebrospinal fluid of children affected with spinal muscular atrophy for further exploration comparing follow-up and baseline phase of therapy.

We obtained RNA samples of patients through the course of their nusinersen therapy and performed miRNA-sequencing to explore which miRNAs are changing throughout therapy. Differential expression analysis comparing follow-up phase to the baseline treatment phase identified 101 miRNAs in the CSF, of which 66 miRNAs were significantly altered. A systematic literature review on the 66 miRNAs identified 15 of these miRNAs that were the most relevant to motor neuron development and pathophysiology (Figure 2). It is important to note that variabilities and transient changes of miRNA expression were observed in individual patient miRNA profiles, and it was the cumulative observation in the 15 miRNAs of a lower expression over the course of treatment. The candidate miRNAs encompassed a spectrum of functions including neuronal maturation, differentiation and growth alongside mediators of motor neuron viability through regulation of apoptotic pathways (Table 2). 

### 3.3. Predicted Target Genes of Differentially Expressed miRNAs Are Shown to Be Involved with Neurogenesis, Neuronal Differentiation, and Growth

**Figure 3:**
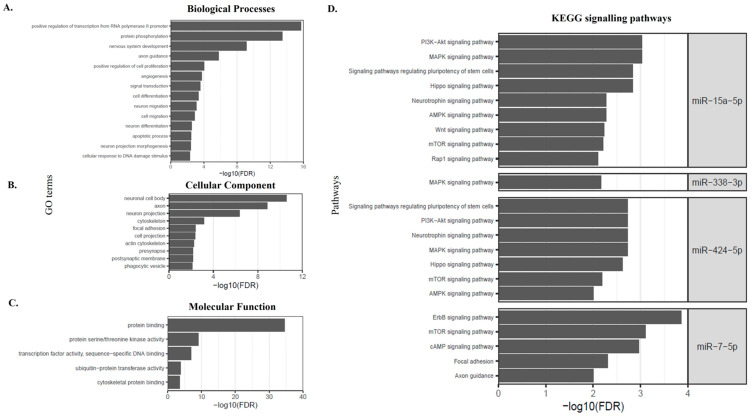
Gene ontology (GO) analysis of the target genes from the 14 candidate miRNAs to elucidate their key roles in (**A**) Biological Processes, (**B**) Cellular Components (**C**) Molecular Function and (**D**) KEGG pathway enrichment analysis of target genes from the 14 candidate miRNAs.

Fourteen of the 15 differentially expressed miRNAs were deemed to fulfil ‘the strong evidence criteria’ from miRTarbase and from at least one other miRNA-target interaction databases (TargetScan and/or miRDB) [17,18,19]. Therefore, these fourteen identified miRNAs were predicted to have the strongest associations with their target genes. The number of target genes predicted for each differentially expressed miRNA varied from 6 (miR-127-3p) to 1244 (miR-126-3p) (mean = 366.71, SD = 377.75). We next performed gene ontology enrichment analysis on the miRNA predicted targeted genes. The analysis of biological processes revealed that the selected miRNAs were mainly involved in regulatory processes, such as regulation of cytoplasmic translation, positive regulation of transcription from RNA polymerase II promoter, protein phosphorylation, nervous system development, axon guidance and neuron projection morphogenesis (Figure 3A). From a cellular component perspective, target genes were involved in neuronal cell body, cytoskeleton, focal adhesion, cell projection (Figure 3B). The molecular function for these target genes included those regulating protein serine/threonine kinase activity, transcriptional factor activity, cytoskeletal protein binding (Figure 3C). 

**Figure 4:**
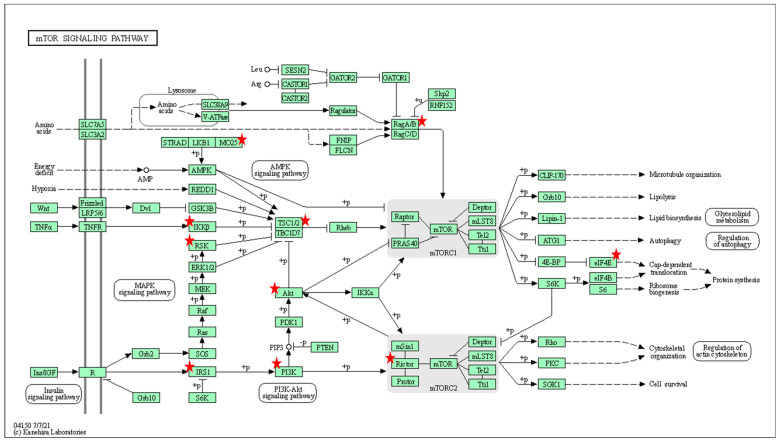
Gene ontology (GO) analysis using the DAVID software. Role of the affected genes (indicated as red stars) within the mTOR signaling pathway associated with miR-378.

**Figure 5:**
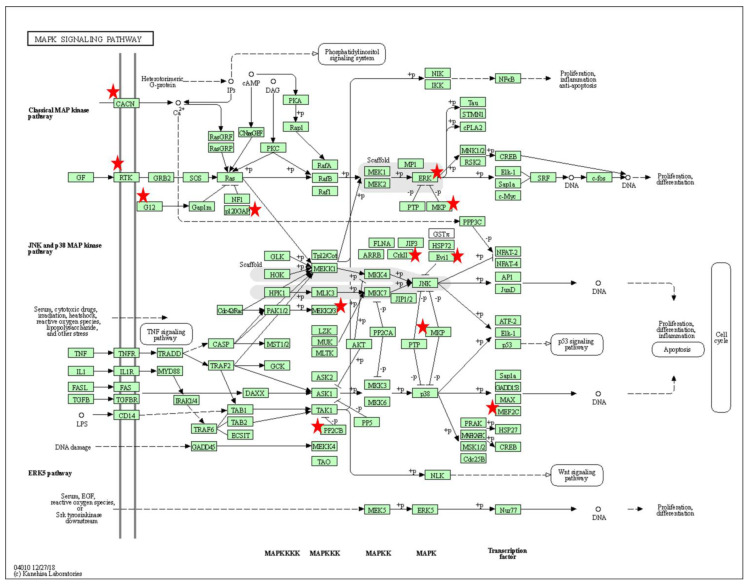
Gene ontology (GO) analysis using the DAVID software. Role of the affected genes (indicated as red stars) within the MAPK signaling pathway associated with miR-338.

Pathway analysis using KEGG (see methods) showed that the putative target genes of the selected miRNAs were associated with discrete and overlapping regulatory and signaling pathways (Table 3, Figure 3D). The signaling pathways that had commonality between two or more miRNAs were involved in cell growth (Mammalian target of rapamycin [mTOR]) (Figure 4), cell adhesion (Rap1 signaling and focal adhesion), metabolic processes (sphingolipid), axon guidance and trophic factors involved in neural cell survival and differentiation (neutrophin signaling pathway). KEGG annotation showed that the target genes predicted to be associated with miR-15a-5p, miR-338-3p and miR-424-5p were significantly enriched (FDR < 0.01) in the mitogen-activated protein kinase (MAPK) signaling pathway (Figure 5). This pathway mediates a wide range of cellular responses, including gene expression, differentiation, proliferation and apoptosis. Furthermore, additional pathways observed to be specific to individual miRNAs included ErbB, Ras, calcium signaling, Cyclic adenosine 3′,5′-monophosphate (cAMP), and phosphatidylinositol signaling pathways amongst others (Table 3).

## 4. Discussion

This exploratory study has for the first time in a paediatric SMA cohort demonstrated that a low-input untargeted transcriptomic profiling methodology is feasible in CSF and can identify alterations in miRNA expression through the course of nusinersen therapy. The techniques used in this study overcome the inherent barriers associated with miRNA analysis in CSF, which is a predominantly acellular biospecimen that reveals a low yield of miRNA. Furthermore, in a therapeutic domain where intrathecal modalities are being continually optimized, identifying a potential biomarker that is specific to the CNS holds potential clinical benefit. 

As the pathogenesis of SMA occurs early in life, with significant denervation occurring in the first three months (16), the miRNA profile of infants with SMA concurrent with developmental progress is particularly vital to ascertain. Furthermore, as the clinical paradigm shifts to timely diagnosis through newborn screening, facilitating access to prompt intervention, the modulation of miRNA, early in disease modification is warranted. Thus, this study uniquely focused on a cohort of children, diagnostically confirmed and treated with nusinersen early in their disease course. Despite our cohort being heterogenous in biogenetic characteristics, an overall downregulation noted in all the identified miRNAs could be secondary to treatment efficacy and/or the changing transcriptomic profile associated with developmental stage. Further longitudinal studies with age matched controls would be essential to delineate these aspects. 

From the current literature, several possible roles of these dysregulated miRNAs in preclinical studies and other motor neurone diseases have been reported. However, the present study is the first to translate this preclinical work and identify patterns of miRNA regulation in vivo, and longitudinally with treatment. From a wide spectrum of possible molecular, biological and cellular pathways, 15 candidate miRNAs identified from this treated cohort were associated with processes of neuronal survival, autophagy/apoptois, neuronal differentiation, and cell signaling. 

### 4.1. Roles of Identified miRNA

#### The Interplay between Neuronal Survival and Autophagy/Apoptotic Pathways

This study identified miR-7 and miR-183, known mediators of autophagy and apoptotic processes, contributing to cell fate decision. 

miR-7, mainly expressed in neurons, reduces neurotoxicity induced apoptosis caused by protein aggregates in neurodegenerative conditions such as Parkinson’s disease, by inhibiting the expression of pro-apoptotic proteins [20]. This miRNA also upregulates the mTOR pathway which is a central signaling pathway involved in promoting cell differentiation and growth. Our study has demonstrated an overall down regulation of miR-7 within the context of SMN repletion administered for on average of one year. From this work, we can postulate that in a lifelong therapy, where functional improvements are seen in the years following treatment [21], there may be a lag time in which miR-7 exerts a neuroprotective role through modification of pro-apoptotic gene expression. 

The balance between cell death and survival is replicated by elucidating the functions of miR-183. This miRNA inhibits axonal growth in motor neurones whilst mediating a protective effect by down regulation of p53, a ubiquitously expressed nuclear protein that controls cell division and cell death [22]. In SMN deficient preclinical models, neurite miR-183 is upregulated. Therefore, its downregulation in SMN repletion as suggested in our study may well be expected. 

### 4.2. miRNAs in the Regulation of Cellular Stress Responses

Neuronal survival and neurite growth are important processes within the development and maturation of the peripheral nervous system. Dysregulation of these normative processes have been noted in peripheral neuropathies such as Charcot-Marie-Tooth type 2 [23]. Heat shock proteins such as Heat Shock Protein Beta-1 (HspB1), have key anti-apoptotic and cytoprotective roles, maintaining the cytoskeletal structure of neurones [24]. With states of physical or metabolic injury, HspB1 are upregulated to ensure that protein folding remains stable [25]. HspB1 particularly enhances neurite growth and axonal regeneration in the peripheral nervous system [26], with these processes mediated by enzymes such as RhoGTPases and RhoA [27]. Heat shock proteins upregulate miR-128. As RhoA-GTPase plays a regulatory role in the organization and stability of cytoskeletal networks through its downstream effectors, miR-128 appears to be the link between HspB1 and maintenance of axonal cytoskeletal structure. In untreated SMA infants, studies have shown overexpression of heat shock proteins secondary to DNA damage, incurred within this SMN deficient environment [28]. Thus, downregulation of miR-128 in our study may represent the amelioration of oxidative stress secondary to SMN repletion by therapeutic intervention, with validation required in larger cohorts.

Neuroinflammation in SMA has been recently proposed as another pathogenic process, leading to the ultimate pathway of neuronal degeneration, and is also well established in other neurodegenerative disorders [29]. This process is mediated by astrocytes, microglia and T cells, which establish a defective immune response. Higher proinflammatory cytokine levels have been noted in both preclinical and patient models in SMA [30]. In our study, a key regulator of the innate immune system, miR-146 was identified. It is known to be activated downstream of cytokine production and Toll-like receptor mediated signaling pathways culminating in NF-Kappa-B transcription (with a ubiquitous role in mediating inflammatory responses). This transcription factor has a central role in a spectrum of neurodegenerative diseases, establishing pro-inflammatory states. As such, its inhibition provides an attractive target to reduce neuronal cell loss through amelioration of these inflammatory processes [31]. Consistent with our data, a study of SMA astrocytes showed increased production and secretion of miR-146 in a knockout SMA mouse model [32]. Moreover, treating immune pluripotent stem cell-derived motor neurons with synthesized miR-146 molecules was sufficient to induce motor neuron loss in vitro [32]. In contrast, miR-146 inhibition prevented SMA astrocyte-induced motor neuron loss. Together, these data indicate that astrocyte upregulation of miR-146 may be a potential mechanism for astrocyte-mediated SMA motor neuron pathology. Deduced from this body of work, nusinersen may ameliorate motor neuron loss by reducing inflammatory states, reflected by deregulation of miR-146. 

### 4.3. The Neurodevelopmental Roles of miRNAs in Neuronal Differentiation

During late neurogenesis and in the early postnatal development, different types of glial cells are generated from neuronal precursor cells. Recent studies support a role for miRNAs in the development of these glial lineages. For example, it has been shown that miR-338 is specifically enriched in oligodendrocyte precursors and plays a functional role in oligodendrocyte differentiation [33]. Thus, miRNAs not only settle major cell fate decisions (e.g., neuronal versus glial differentiation), but also determine the expression of specific glial and neural cell types, for example whether a neuron becomes a motor neuron versus an interneuron. 

Whilst our prior understanding of SMA centered on the survival of the alpha motor neurone and secondary effects on muscle morphology and function, molecular technologies have generated new knowledge attesting to the roles of miRNAs in non-motor neurone tissues in SMA pathogenesis. Particularly, SMN has a key role in myogenesis and mitochondrial biogenesis, with histopathologic features of SMA consistent with aberration of these normative processes [34]. Interestingly, our study demonstrated reduced levels of miR-378 in longitudinal CSF specimens from treated SMA individuals. miR-378a-3p has a key role in myogenesis and its overexpression in animal models has been associated with regulation of skeletal muscle growth by promoting myoblast differentiation and inhibiting proliferation [35]. The pathways leading to dysregulation of these processes has been evaluated in preclinical models where miR-378 deficiency is accompanied by accumulation of abnormal mitochondria, and excessive apoptosis in skeletal muscle, leading to atrophy of skeletal muscle bulk, whereas repletion reverses these processes [36]. miR-378 appears to be a key signaling miRNA involved in pathways that regulate the balance between myocyte autophagy and apoptosis, the former through the mTOR pathway and the latter through targeting Caspase 9. These studies highlight a vital role of miR-378 in maintaining normal muscle homeostasis. The persistent down regulation of miR-378 in our study, despite therapeutic intervention may speak to the CNS targeted administration of nusinersen, which precludes SMN repletion to tissues beyond the nervous system.

miRNAs, particularly miR-196 as found in the present study, are involved in the precise regulation of Hox genes in a spatial and temporal manner during development [37]. Hox genes play an essential role in the spatial patterning of neurons within brain and spinal cord tissue. Expression of miR-196 at a specific time during development is required for normal motor neuron differentiation in chick embryos via down-regulation of Homeobox B8 (Hoxb8). Failure to clear Hoxb8 in a spatial and temporal manner leads to the abolition of motor neuron genesis within the neural tube leaving cells in a neural progenitor cell state [38]. However, the inhibition of miR-196 alone does not lead to continual expression of Hoxb8 in the neural tube, suggesting the possibility of other miRNAs working together with miR-196 to silence Hoxb8 expression and promote motor neuron development [39]. Whilst clinical studies are awaited, this evidence provides a mechanistic foundation that aligns with the hypothesis that SMA is a neurodevelopmental as well as neurodegenerative disease mediated at least in part by the complex interactions of miRNA [8]. Our miRNA-seq findings mirror electrophysiological studies that show that these two concurrent processes are occurring (neurodegeneration) and dysregulated (neurodevelopment) especially early in the CNS maturation profile of children with SMA [8,9]. 

### 4.4. miRNAs in the Context of Cell Signaling 

Whilst individual miRNAs have been identified through this study, the complexity and variety of their roles in disease mechanisms and disease modifying processes involves interrogation of the signaling pathways that they mediate. 

The MAPK pathway has been identified in this study as a dominant process in treated SMA, with miR-15a-5p and miR-338-3p amongst the miRNAs facilitating its translation. MAPK is a fundamental stress-activated signal transduction process that regulates differentiation, proliferation, cell death, survival, and transformation. The pathway is activated by a wide range of stimuli, including oxidative stress. Many studies have shown that signaling is overactive in amyotrophic lateral sclerosis patients. Inhibiting MAPK signaling protects neurons from degeneration [40]. Similarly, in the context of SMA, in vivo studies have reported activation of the MAPK signaling pathway as a response to negative regulation of *SMN2* expression [41]. Cumulatively, our study proposes that downregulation of miRNA mediators with SMN repletion reduces MAPK mediated mitogen- and stress-activated signals to ameliorate neuronal injury.

Another dominant signaling pathway highlighted by our study was the mTOR pathway, proposed in this study to be mediated by miR-183 and miR-378. Upstream components of this pathway are mediated by phosphatidylinositol 3 kinase (associated with identified miR-7-5p, miR-15a-5p, miR-424-5p). Studies have reported reduced mTOR activity and impaired protein synthesis [42]. The mTOR signaling pathway regulates gene transcription and protein synthesis to regulate cell proliferation and immune cell differentiation. A recent study confirmed that via the mTOR pathway, C1q/TNF-related protein 3 (CTRP3) enhances axonal outgrowth and regulates SMN protein synthesis, which are well-known impaired processes in SMA motor neurons [43]. Whilst therapeutics focusing on the regulation of mTOR are already within clinical practice, unravelling the complex roles of miRNAs associated with mTOR pathways specific to the pathogenies of SMA may reveal novel strategies for intervention. 

## 5. Limitations and Future Directions

Due to the limits of cohort size, we were unable to address the clinical and genetic confounders of miRNA expression in treated SMA. However, this study was specifically conducted to ascertain a selection of target miRNAs that could be validated in a wider cohort of children with a spectrum of disease durations, phenotype and genotype in future studies. This will enable us to understand the individualistic roles whilst unravelling the complexities of miRNA functions as highlighted in the current study. Furthermore, and unique to a paediatric onset disease, miRNAs profiles within a physiological developmental window should be interrogated in future studies with incorporation of age matched controls. This will enable delineation of whether the changes seen in this study are wholly due to therapeutic response, residual disease mechanisms, developmental processes, or combinations of these factors. This exploratory study has shown for the first time that miRNAs can be effectively identified in CSF of treated children with SMA. Due to the versatile roles of miRNA in regulating cell survival, differentiation and development as identified in this study, it is proposed that alterations in miRNA can give a holistic view of disease mechanisms alongside processes contributing to disease modification with treatment, enabling the next frontier of biomarker development in SMA. 

## Figures and Tables

**Table 1:** Clinical characteristics of study participants undergoing nusinersen therapy.

Patient	Gender	*SMN2* Copy Number	Age at Diagnostic Confirmation (Days)	Age at Therapeutic Intervention (Days)	Age at Collection of First CSF Specimen (Baseline) (Days)	Age at Collection of Second CSF Specimen (Follow-Up) (Days)	Disease Duration at Treatment Initiation (Days)	Function (CHOP-INTEND)	
								Baseline	Follow-Up
1	Male	2	13	22	80	438	7	37	58
2	Female	2	365	390	390	615	61	45	48
3	Female	3	10	33	101	339	6	59	NA
4	Male	2	16	16	16	206	7	27	54
5	Female	3	7	25	25	208	0	61	64
6	Male	2	9	37	52	100	0	64	NA

Data are represented as number of subjects. Disease duration is the interval between age of symptom onset and age at first nusinersen treatment. CSF; cerebrospinal fluid. *SMN2*; survival of motor neuron 2. CHOP-INTEND; The Children’s Hospital of Philadelphia Infant Test of Neuromuscular Disorders.

**Table 2:** Main functions of the differentially expressed miRNAs in SMA with nusinersen therapy.

miRNA	Log Fold Change	*p*-Value	Function	PubMed Unique Identifier
miR-7-5p	−3.22	1.47 × 10^−8^	Negative regulation of sprouting angiogenesis, mRNA binding involved in posttranscriptional gene silencing	27431648, 31501273
miR-15a-5p	−3.25	5.22 × 10^−5^	Enhances cell viability and inhibits apoptosis	32384924
miR-15b-5p	−3.63	0.0003	Promotes neurogenesis and inhibits neural progenitor proliferation promotes proliferation, decreases apoptosis	20584895 32165184
miR-15b-3p	−2.67	4.18 × 10^−9^		
miR-126-5p	−2.32	0.0005	Promotes angiogenesis and neurogenesis, master regulator of NMJ function, Linked to neuronal loss, neurodegeneration and apoptosis in primary cultured spinal neurones	29773756 29773756 27748416
miR-127-3p	−1.87	0.0001	Activate autophagy in the cortical neurons	33723216
miR-130a-3p	−2.43	1.35 × 10^−7^	Regulate neurotransmitter synthesis	17855557
miR-146b-5p	−1.81	8.84 × 10^−5^	Motor neuron loss caused by astrocyte-mediated pathology through NFkB signaling	28637335
miR-183-5p	−1.76	1.04 × 10^−5^	Protein synthesis; axonal outgrowth	29160009, 24523674, 25055867, 26459109
miR-196b-5p	−1.30	0.0022	Confines the rostrocaudal axis in the neural tube	20553899
miR-324-3p	−1.20	9.28 × 10^−5^	Promotes neuronal differentiation and neurite outgrowth	23527072
miR-338-3p	−1.52	5.54 × 10^−8^	Acts as a negative regulator of neuronal differentiation by suppressing apoptosis-associated tyrosine kinase and cytochrome oxidase complex IV	19020050 18684991
miR-378a-3p	−1.59	0.0003	Promotes differentiation and inhibits proliferation of myoblasts in skeletal muscle development	27661135
miR-424-5p	−1.88	8.65 × 10^−6^	Suppress microglia activation, regulation of cellular activities via interacting with specific lncRNA sponges	32065781

The filtering was informed by a comprehensive literature search using a constellation of single and combined key search terms in PubMed including ‘motor neuron development’, ‘neurodegeneration’, ‘spinal muscular atrophy’, ‘survival motor neuron protein’ and ‘nusinersen’. A negative log fold change indicates downregulation of miRNA in the follow-up phase of nusinersen therapy.

**Table 3:** KEGG pathway of predicted target genes of the differentially expressed miRNAs. Key pathways have been included in this table.

miRNA	Pathways	Target Genes	FDR
miR-7-5p	ErbB signaling pathway	CAMK2D, PAK1, RPS6KB1, ERBB4, PRKCB, AKT3, PIK3R3, PIK3CD, RAF1, PAK2, PTK2, EGFR	1.38 × 10^−4^
	mTOR signaling pathway	RPS6KB1, IRS1, PRKCB, AKT3, DDIT4, PIK3R3, ULK2, PIK3CD, EIF4E	7.76 × 10^−4^
	cAMP signaling pathway	CAMK2D, PDE4D, PIK3CD, RRAS2, PIK3R3, ATP2B2, GLI3, RELA, PAK1, GRIN2A, ADCY9, AKT3, PDE4B, PDE4A, RAF1	0.001
	Focal adhesion	PRKCB, XIAP, PIK3CD, PIK3R3, PARVA, EGFR, PTK2, IGF1R, PAK1, COL2A1, AKT3, RAF1, PAK2, ITGA9	0.004
	Calcium signaling pathway	ITPKC, CAMK2D, GRIN2A, ADCY9, ERBB4, PRKCB, PPIF, VDAC3, VDAC1, ATP2B2, PLCB1, EGFR	0.009
	Axon guidance	PAK1, DPYSL2, SEMA4C, SLIT1, PLXNA1, NFATC2, SRGAP2, PAK2, PTK2, EPHA3	0.009
miR-15a-5p	PI3K-Akt signaling pathway	CSF1, IRS1, LAMC1, PIK3R1, FGF2, IGF1R, GHR, IKBKB, CCND3, FGF7, RELN, CCND2, FGF9, PPP2R1B, CCND1, YWHAQ, PPP2R1A, AKT3, MYB, EIF4E, YWHAH, MAP2K1, COL24A1, CHUK, INSR, TSC1, PPP2R5C, VEGFA, CDK6, CCNE1, ITGA10, FGF18, BCL2, RAF1, SGK1, SOS2, FGFR1	9.43 × 10^−4^
	MAPK signaling pathway	PTPRR, FGF2, CACNA1E, CRKL, ELK4, IKBKB, RPS6KA3, RPS6KA6, FGF7, MAPK8, FGF9, MKNK1, GNA12, AKT3, MAP3K4, MAP2K3, MAP2K1, CHUK, BDNF, CACNA2D1, NFATC3, PPM1A, MRAS, TAOK1, FGF18, NF1, RAF1, SOS2, HSPA1B, FGFR1	9.43 × 10^−4^
	Hippo signaling pathway	YAP1, WNT2B, WNT3A, WWC1, FZD6, WNT7A, AXIN2, AMOT, CCND3, PARD6B, LATS2, CCND2, PPP2R1B, CCND1, YWHAQ, PPP2R1A, BTRC, TEAD1, YWHAH, BMPR1A, WNT4	0.001
	Signaling pathways regulating pluripotency of stem cells	MAP2K1, ZFHX3, WNT2B, WNT3A, FZD6, WNT7A, PIK3R1, AXIN2, FGF2, ACVR2B, ACVR2A, IGF1R, AKT3, OTX1, RAF1, JARID2, SKIL, BMPR1A, FGFR1, WNT4	0.001
	Neurotrophin signaling pathway	MAP2K1, PRDM4, BDNF, IRS1, FRS2, PIK3R1, CRKL, IKBKB, RPS6KA3, RPS6KA6, MAPK8, IRAK2, ARHGDIA, AKT3, BCL2, RAF1, SOS2	0.005
	AMPK signaling pathway	CAB39, IRS1, STRADB, INSR, TSC1, PIK3R1, PPP2R5C, ACACB, FOXO1, IGF1R, RAB10, LIPE, PPP2R1B, CCND1, PPP2R1A, FASN, AKT3	0.005
	Wnt signaling pathway	WNT2B, ROCK2, WNT3A, FZD6, SIAH1, NFATC3, WNT7A, AXIN2, NKD1, LRP6, FOSL1, CCND3, MAPK8, CCND2, CCND1, TBL1XR1, BTRC, WNT4	0.005
	mTOR signaling pathway	IKBKB, RPS6KA3, RPS6KA6, RRAGA, CAB39, IRS1, AKT3, TSC1, RICTOR, PIK3R1, EIF4E	0.006
	Rap1 signaling pathway	MAP2K3, MAP2K1, CSF1, INSR, GNAI3, PIK3R1, SIPA1L2, FGF2, ADCY5, CRKL, IGF1R, VEGFA, PARD6B, FGF7, MRAS, ADORA2A, FGF9, RASSF5, GNAQ, AKT3, FGF18, RAF1, FGFR1	0.007
miR-338-3p	MAPK signaling pathway	MAP3K2, MEF2C, PPM1B, CACNB4, MECOM, RASA1, CACNA2D1, MAPK1, GNG12, CRK, DUSP16, FGFR2	0.006
miR-424-5p	MAPK signaling pathway	PTPRR, FGF2, CACNA1E, CRKL, ELK4, IKBKB, RPS6KA3, RPS6KA6, FGF7, MAPK8, FGF9, MKNK1, GNA12, AKT3, MAP3K4, MAP2K3, MAP3K3, MAP2K1, BDNF, CACNA2D1, NFATC3, PPM1A, MRAS, TAOK1, FGF18, NF1, RAF1, SOS2, FGFR1	0.001
	Signaling pathways regulating pluripotency of stem cells	MAP2K1, ZFHX3, WNT2B, WNT3A, FZD6, WNT7A, PIK3R1, AXIN2, FGF2, ACVR2B, ACVR2A, IGF1R, AKT3, OTX1, RAF1, JARID2, SKIL, BMPR1A, FGFR1, WNT4	0.001
	PI3K-Akt signaling pathway	IRS1, LAMC1, PIK3R1, FGF2, IGF1R, GHR, IKBKB, CCND3, FGF7, RELN, CCND2, FGF9, PPP2R1B, CCND1, YWHAQ, PPP2R1A, AKT3, MYB, EIF4E, YWHAH, MAP2K1, COL24A1, INSR, TSC1, PPP2R5C, VEGFA, CDK6, CCNE1, ITGA10, FGF18, BCL2, RAF1, SGK1, SOS2, FGFR1	0.001
	Neurotrophin signaling pathway	MAP3K3, MAP2K1, PRDM4, BDNF, IRS1, FRS2, PIK3R1, CRKL, IKBKB, RPS6KA3, RPS6KA6, MAPK8, IRAK2, ARHGDIA, AKT3, BCL2, RAF1, SOS2	0.001
	Hippo signaling pathway	YAP1, WNT2B, WNT3A, WWC1, FZD6, WNT7A, AXIN2, AMOT, CCND3, PARD6B, LATS2, CCND2, PPP2R1B, CCND1, YWHAQ, PPP2R1A, BTRC, YWHAH, BMPR1A, WNT4	0.002
	mTOR signaling pathway	IKBKB, RPS6KA3, RPS6KA6, RRAGA, CAB39, IRS1, AKT3, TSC1, RICTOR, PIK3R1, EIF4E	0.006
	AMPK signaling pathway	CAB39, IRS1, STRADB, INSR, TSC1, PIK3R1, PPP2R5C, ACACB, IGF1R, RAB10, LIPE, PPP2R1B, CCND1, PPP2R1A, FASN, AKT3	0.009

## Data Availability

The data presented in this study are available on request from the corresponding author.
